# Hypothalamic POMC Neurons Gate the Fear‐Feeding Trade‐Off

**DOI:** 10.1002/advs.78379

**Published:** 2026-10-20

**Authors:** Cristina Miralpeix, Abel Eraso‐Pichot, Vincent Simon, Victor Jouque, Melissa Sadallah, Samantha Clark, Philippe Zizzari, Urszula Skupio, Ana Covelo, Nathalie Dupuy, Jean Charles Nicolas, Astrid Cannich, Thierry Leste‐Lasserre, Guillaume Laplagne, Carmelo Quarta, Giovanni Marsicano, Daniela Cota

**Affiliations:** ^1^ University of Bordeaux INSERM Neurocentre Magendie U1215 Bordeaux France

**Keywords:** CB1R, competing behaviors, CRH, fear, feeding, HPA axis, POMC

## Abstract

To survive, animals must constantly balance the need to obtain food with the need to avoid danger. Feeding is essential, yet perceived danger shifts priority toward safety and transiently suppresses food intake, even when organisms are hungry. Hypothalamic pro‐opiomelanocortin (POMC) neurons align metabolic outputs with energy demands and control feeding. However, the role of POMC neurons in determining the trade‐off between feeding and threat responses remains unclear. Here, fasted mice are exposed to a fear‐conditioning paradigm to examine how POMC neurons influence the choice between feeding and defensive behaviors. Behavioral analyses combined with chemogenetic, neuroanatomical, and molecular approaches show that activation of POMC neurons suppresses feeding during a learned threat and it is associated with corticotropin releasing hormone (CRH) neuron–dependent activation of the hypothalamic–pituitary–adrenal (HPA) axis. These effects require cannabinoid type 1 receptor (CB1R) signaling in POMC neurons, uncovering a POMC–CB1R pathway that tunes behavioral flexibility under a perceived threat. Collectively, the data identify POMC neurons as key integrators of metabolic and stress signals that bias decision making between feeding and threat‐avoidance behaviors.

## Graphical abstract

Hungry animals must continually arbitrate between feeding and defensive behaviors when confronted with potential threats. We show that hypothalamic POMC neurons engage CRH‐expressing stress circuits and downstream hormonal responses to coordinate this behavioral selection between competing behaviors. CB1 receptor signaling in POMC neurons modulates this process, biasing behavior toward defensive responses over feeding.

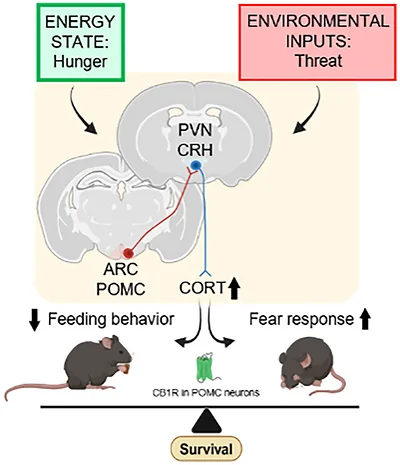

## 1 Introduction

The decision to eat is fundamental to survival, yet its adaptive value depends on context [1, 2, 3]. In safe environments, feeding is prioritized to meet energy and nutrient requirements. In contrast, when a threat is perceived, defensive responses override feeding, even in hungry states, prioritizing immediate safety over ingestion [3, 4, 5, 6, 7]. Thus, optimal expression of feeding behavior requires adaptive control mechanisms that continuously integrate internal energy needs with changing environmental conditions [2]. These processes are expected to require rapid modulation of autonomic and endocrine responses, particularly the regulation of the hypothalamic‐pituitary‐adrenal (HPA) axis by corticotropin‐releasing hormone (CRH) neurons in the hypothalamic paraventricular nucleus (PVN), which, in turn, affects behavioral outcomes [1, 8, 9]. Notwithstanding, the underlying mechanisms coupling the rapid prioritization of competing needs, namely hunger vs. facing a threat, remain poorly defined.

Hypothalamic pro‐opiomelanocortin (POMC) expressing neurons align whole‐body metabolic responses with energy supply and demand through the autonomic nervous system, the endocrine pituitary, and the control of food intake [10, 11]. POMC neurons express cannabinoid type 1 receptors (CB1R) —key modulators of neuronal activity and neurotransmitter release— whose signaling tunes POMC neuronal function with the internal energy state [12, 13]. Beyond metabolic control, recent studies suggest that POMC neurons can contribute selectively to behavioral responses to stress. For example, they are engaged by physical restraint, but not by other stressors [10, 11, 14], suggesting that they may integrate internal metabolic state with specific environmental challenges. However, whether POMC neurons and CB1R contribute to the behavioral trade‐off between fear and feeding that optimizes survival remains unexplored.

In this work, by combining acute behavioral analyses of the transition from defensive behavior to feeding with chemogenetic, neuroanatomical and molecular approaches, we show that activation of POMC neurons suppresses the resumption of feeding in fasted mice during learned‐threat retrieval. This is associated with CRH neuron‐dependent activation of the HPA axis. Interestingly, CB1R in POMC neurons modulates these responses, establishing a novel mechanism that governs survival‐related decision making between feeding and threat‐avoidance.

## 2 Results

### 2.1 Conditioned Fear Suppresses Feeding in Fasted Mice

To simultaneously assess neuronal mechanisms underlying behavioral prioritization between feeding and fear‐related responses, we adapted a previously described associative learning paradigm in which fearful stimuli suppress feeding [15] (Figure 1A). To this end, C57BL/6N mice were conditioned with three tones paired with foot‐shocks (“Conditioned”, abbreviated as “Cond”) or not paired (“Unconditioned”, abbreviated as “Uncond”). Following conditioning, mice were fasted for 24 h and placed in a novel environment containing a food pellet. In the novel context, mice were allowed to explore the environment, and the latency until the first bite to the food pellet was measured (latency to eat before tone). Upon this first bite, the tone was presented to assess food‐seeking behavior (latency to eat after tone) (Figure 1A). This protocol has the advantage to measure the latency to eat in fasted animals before and after the threat (the tone associated with the foot‐shocks) is presented, as well as the total food intake, the time eating and the fear‐related response (time immobile) (Figure 1A).

**FIGURE 1:**
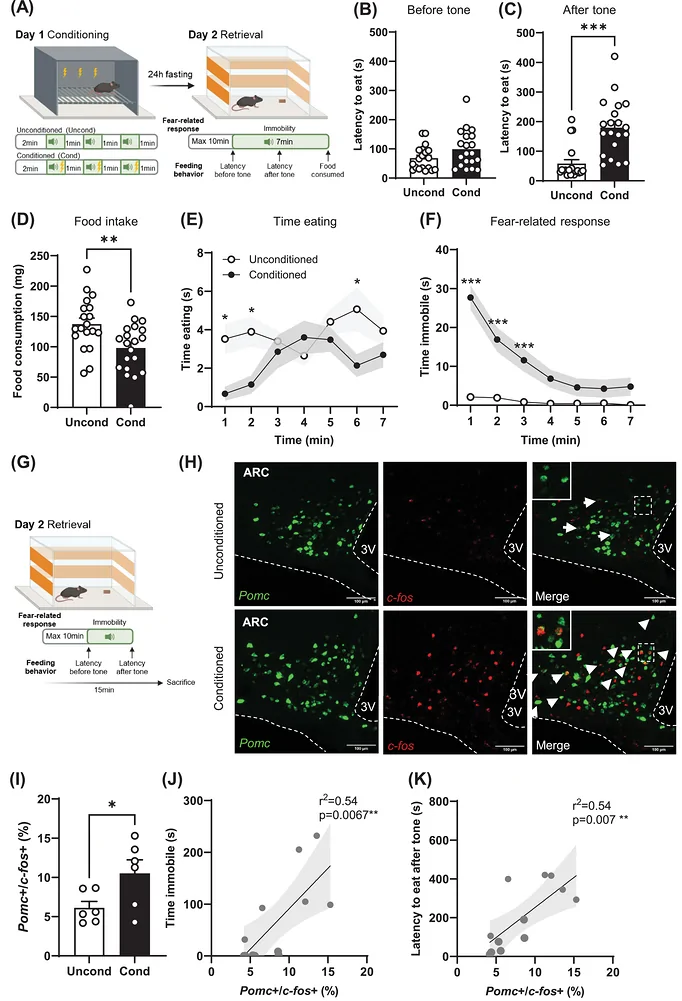
Fear suppresses feeding in fasted mice and activates hypothalamic POMC neurons. (A) Fear‐suppression of feeding paradigm in fasted mice. (B, C) Conditioned mice in day 1, followed by a 24 h fasting show reduced latency to eat after (C), but not before (B) the tone was played in day 2 (retrieval) (*n*  =  19–20 mice/group). (D) Total food consumption. (E) Time spent eating is reduced in conditioned mice during the first minutes of the test, while (F) the time spent immobile was increased. (G) The fear‐suppression of feeding paradigm was modified to stop the test at the latency to eat after tone, and mice were sacrificed 15 min after the beginning of the test. (H) Representative images of FISH for *Pomc* and *c‐fos* in C57BL/6J male mice (*Pomc*, green; *c‐fos*, red). Scale bars, 100 µm. (I) Conditioned mice have increased *c‐fos* mRNA expression in *Pomc*‐expressing cells after retrieval (*n*  =  6 mice/group). (J) *c‐fos* in *Pomc*‐expressing neurons positively correlates with the time mice spent immobile (*n*  = 12 mice) and (K) with the latency to eat after the tone (*n*  =  12 mice). An unpaired two‐tailed Student's *t*‐test was performed when comparing two conditions in (B–D, G; repeated‐measures two‐way ANOVA was applied to E and F; two‐tailed Pearson correlation was used in (H, I). Data are represented as the mean ± s.e.m. ^*^ *p* ≤ 0.05, ^**^ *p* ≤ 0.01, ^***^ *p* ≤ 0.001. ARC: arcuate nucleus of the hypothalamus; 3V: third ventricle. Panel A was created using BioRender.com.

As expected, while the latency to eat before the tone did not differ between groups (Figure 1B), conditioned mice had increased latency to eat after the tone compared to unconditioned animals (Figure 1C). During a 7‐min test with a continuous tone, conditioned mice showed significantly decreased food intake (Figure 1D), with this difference primarily occurring during the first 2 min of the tone presentation (Figure 1E). Likewise, regarding the fear‐related response, mice in the conditioned group exhibited immobility only during the initial 3 min of tone presentation (Figure 1F). These data demonstrate that induction of a fear‐related state reduces feeding behavior and increases immobility response in fasted mice, consistent with previous reports [15], thereby validating this modified behavioral paradigm.

### 2.2 Hypothalamic POMC Neurons are Activated in Fasted Mice Confronted With a Behavioral Trade‐Off Between Fear and Feeding

Hypothalamic POMC neurons classically reduce food intake through melanocortin pathways [10, 11]. Hence, we examined whether POMC neurons were differentially activated in conditioned and unconditioned mice. Since the changes in feeding behavior due to a threat occurred during the first minutes of the test (Figure 1E,F) and POMC neurons are rapidly activated upon food presentation and consumption [16], we assessed the mRNA expression of the early activation marker *c‐fos* in POMC neurons in animals sacrificed 15 min after the start of the test, during the latency‐to‐eat phase, immediately after tone presentation (Figure 1G). As compared to unconditioned animals, conditioned mice displayed increased c‐*fos* mRNA expression in POMC neurons (Figure 1H,I), without changes in their number (Figure S1A). Similarly, c‐Fos protein levels assessed 90 min after the start of the test (Figure S1B) were increased in conditioned mice, without changes in the number of POMC‐expressing neurons (Figure S1C). Notably, c‐*fos* mRNA expression in POMC neurons directly correlated with the immobility time (Figure 1J) and the latency to eat after the tone (Figure 1K), suggesting that POMC neuron activation is associated to the fear‐induced suppression of feeding.

### 2.3 POMC Activation is Necessary for Fear‐Suppression of Feeding

To establish the necessity of POMC neuron activation for the observed behavior, an adeno‐associated viral vector (AAV) encoding a Cre‐dependent Gi‐coupled inhibitory “Designer Receptors Exclusively Activated by Designer Drugs” (DREADD) expressing a modified form of the human muscarinic receptor (hM4Di) was bilaterally injected into the hypothalamic arcuate nucleus (ARC) of POMC^Cre^ mice (Figure S2A), which were then subjected to conditioning (Figure 2A). As expected, injection of the DREADD ligand clozapine N‐oxide (CNO) to hM4Di‐expressing POMC mice significantly reduced POMC neurons activation during the test (Figure 2B), without altering the total number of *Pomc* mRNA‐expressing cells (Figure S2B) or the latency to eat before the tone (Figure 2C). Strikingly, chemogenetic inhibition of POMC neurons reduced both the latency to eat after the tone (Figure 2D) and the freezing response (Figure 2E). In control experiments, CNO administered to mice not expressing DREADDs did not alter any of the analyzed parameters (Figure S2C–F), including food intake (Figure S2G). Together, these data indicate that hypothalamic POMC neurons play a causal role in integrating feeding and fear‐related states.

**FIGURE 2:**
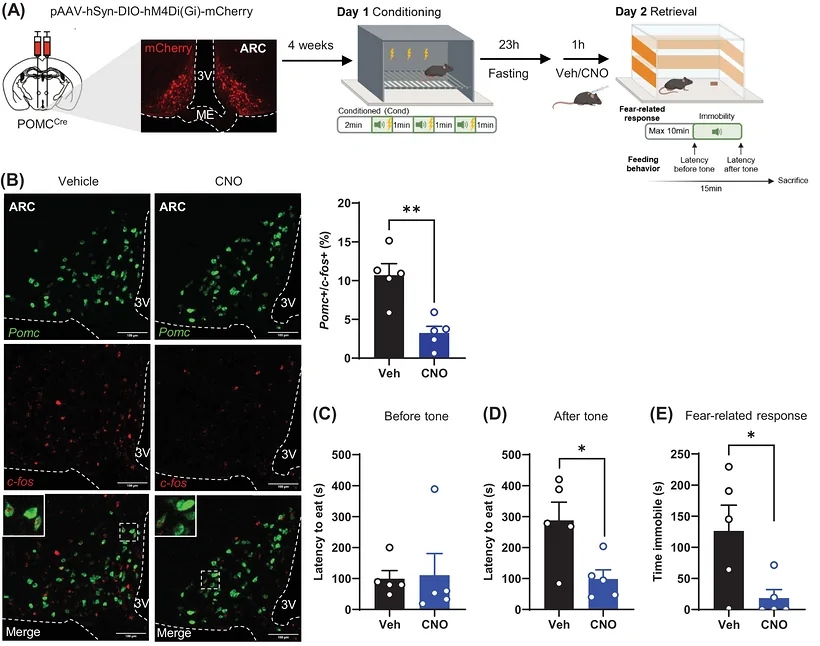
Inhibition of hypothalamic POMC neurons impairs fear‐suppression of feeding. (A) Schematic of AAV‐based administration and expression in the ARC of POMC^Cre^ mice encoding for inhibitory DREADDs (hM4Di) and the associated behavioral paradigm used. (B) CNO injection decreased *c‐fos* mRNA expression in POMC neurons of the ARC. Representative images of FISH for *Pomc* and *c‐fos* in POMC^Cre^ mice (*Pomc*, green; *c‐fos*, red). Scale bars, 100 µm. (C–E) Latency to eat before the tone was not altered by inhibiting POMC neurons (C), however, after the tone, CNO administration decreased latency to eat (D) and fear response (E) (*n* = 5 mice/group). Data are represented as the mean ± s.e.m. Unpaired two‐tailed Student's *t*‐test. ^*^ *p* ≤ 0.05, ^**^ *p* ≤ 0.01. ARC: arcuate nucleus of the hypothalamus; 3V: third ventricle.

### 2.4 Hypothalamic CRH Neurons are Recruited During Fear‐Suppression of Feeding

Next, we wondered how POMC neuronal activation could modulate feeding in response to a threat. Recent studies have demonstrated that hypothalamic POMC neurons send projections to CRH‐expressing neurons in the PVN, orchestrating the endocrine response to specific stress stimuli, such as physical restraint [14, 17]. As CRH neurons trigger the classical stress response through the activation of the HPA axis [8], we hypothesized that this pathway may contribute to the regulation of competing feeding and fear‐related behaviors. We found that conditioned mice exhibited 90% of *Crh*‐positive neurons co‐expressing *c‐fos* mRNA, compared to 30% in unconditioned animals (Figure 3A), with no changes in the number of *Crh*‐positive neurons (Figure S3A). Importantly, *c‐fos* levels in *Crh*‐positive neurons in the PVN positively correlated with those of *Pomc‐*expressing neurons in the ARC (Figure 3B), suggesting their coordinated recruitment during fear‐induced suppression of feeding.

**FIGURE 3:**
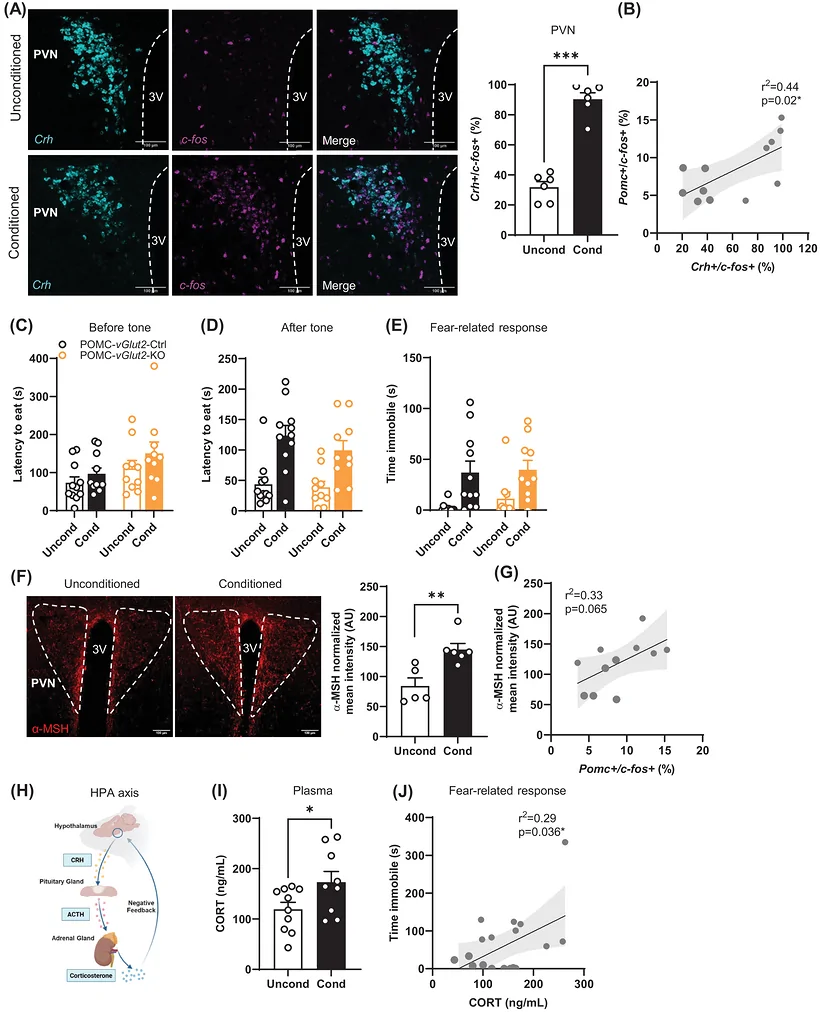
Fear‐suppression of feeding activates hypothalamic CRH neurons and increases corticosterone plasma levels. (A) Representative images of FISH for *Crh* and *c‐fos* mRNA expression in mice after the test (*Crh*, turquoise; *c‐fos*, magenta). Scale bars, 100 µm. Conditioned mice had increased *c‐fos* mRNA expression in *Crh‐*positive cells after the retrieval (*n*  =  6 mice/group). (B) *c‐fos* expression in *Crh*‐expressing neurons positively correlates with the percentage of *c‐fos* in *Pomc*‐expressing cells (*n*  =  12 mice). Blocking glutamate release from POMC neurons does not alter fear‐suppression of feeding behavior since conditioned POMC‐*vGlut2*‐KO mice did not present any difference in the latency to eat before (C) or after the tone (D), nor did they show changes in immobility time compared to their conditioned control littermates (E) (*n* = 10 – 11 mice/group). Both latency to eat after the tone (D) and the fear‐related response (E) show a statistically significant conditioning effect (*p*<0.0001 in both data sets), independent of genotype. (F) Representative images of α‐MSH immunohistochemistry in the PVN of C57BL/6N mice. Conditioned mice had increased α‐MSH intensity in the PVN (*n* = 5–6 mice/group). (G) α‐MSH intensity tended to positively correlate with *c‐fos* in *Pomc*‐expressing neurons. (H) Schematic diagram illustrating the HPA axis. CRH initiates the stress response, modulating ACTH levels produced by the pituitary and CORT levels released by the adrenal glands. (I) As compared to the unconditioned group, conditioned mice have increased corticosterone (CORT) plasma levels (*n*  =  9–10 mice/group). (J) Increased plasma CORT in conditioned mice correlated with the time immobile (*n*  =  9–10 mice/group). Unpaired two‐tailed Student's *t*‐test was performed when comparing two conditions in (A, F, I); two‐way ANOVA was applied to (C–E); two‐tailed Pearson correlation was used in (B, G, J). Data are represented as the mean ± s.e.m. ^*^ *p* ≤ 0.05, ^**^ *p* ≤ 0.01, ^***^ *p* ≤ 0.001. ARC: arcuate nucleus of the hypothalamus, PVN: paraventricular nucleus of the hypothalamus, 3V: third ventricle.

### 2.5 α‐MSH But Not Glutamate Released From POMC Neurons is Involved in the Behavioral Trade‐Off Between Fear and Feeding

POMC neurons are heterogeneous and can release γ‐aminobutyric acid (GABA), glutamate, or both neurotransmitters [10, 12, 18, 19]. As glutamatergic POMC neurons seem to be responsible for the signaling onto CRH neurons during physical restraint [17], we first investigated whether these ARC POMC‐PVN excitatory inputs might contribute to the acute shift between fear and feeding behaviors. To test this possibility, we used mutant mice carrying the inducible deletion of the vesicular glutamate transporter 2 gene (*vGlut2*; gene *Slc17a6*) in POMC neurons after tamoxifen administration (POMC^CreERT2^;vGlut2^fl/fl^ hereafter called POMC‐*vGlut2*‐KO; Figure S3B,C) which suppresses glutamate release [20, 21]. Both unconditioned and conditioned POMC‐*vGlut2*‐KO mice behaved similarly to their POMC‐*vGlut2*‐control littermates, concerning the latency to eat before (Figure 3C) and after the tone (Figure 3D) and in the fear‐related response (Figure 3E). Thus, POMC neurons are very unlikely to modulate fear‐suppression of feeding through the release of glutamate.

Besides neurotransmitters, hypothalamic POMC neurons can communicate through the melanocortin neuropeptide α‐melanocyte‐stimulating hormone (α‐MSH), a product of the cleavage of the POMC peptide that inhibits food intake [10, 22, 23, 24]. CRH neurons express the α‐MSH receptor melanocortin receptor type 4 (MC4R) and can be activated by melanocortin agonists [25, 26, 27], providing a potential direct mechanism for integrating metabolic needs with a stress response. As compared to unconditioned animals, α‐MSH immunoreactivity was increased in the PVN of conditioned mice (Figure 3F) and α‐MSH intensity tended to directly correlate with *c‐fos* mRNA levels in POMC neurons (Figure 3G), suggesting a probable increase of α‐MSH signaling onto the PVN during fear‐suppression of feeding. Hence, the lack of phenotype in POMC‐*vGlut2*‐KO and the increase in α‐MSH support the observation that fast satiety pathways do not involve POMC glutamate release, but instead likely rely on α‐MSH signaling to ensure rapid suppression of feeding [21].

### 2.6 Fear‐Suppression of Feeding is Associated With Increased Corticosterone Levels

Our results suggest that POMC neurons release α‐MSH to activate CRH neurons which, in turn, activate the HPA axis, a key hormonal cascade that orchestrates whole‐body adaptations to stressors, largely via increased corticosterone (CORT) release from the adrenal glands [28] (Figure 3H). Accordingly, we measured circulating CORT levels and found that conditioned mice displayed elevated plasma CORT compared to unconditioned control mice (Figure 3I). These hormonal changes correlated with the fear‐related behavioral response (Figure 3J), indicating an association between HPA‐axis activation and the magnitude of the behavioral response to the threat.

### 2.7 CB1R in POMC Neurons Tune Fear and Feeding Behaviors

We therefore went on to investigate potential molecular mechanisms regulating POMC neuron activation in fear‐suppression of feeding and specifically asked whether CB1R signaling in POMC neurons contributes to the transition from fear‐related behavior to feeding. This hypothesis was motivated by our prior findings that hypothalamic α‐MSH levels are inversely related to CB1R signaling in POMC neurons under stimulated food intake [12, 13], together with evidence that CB1R regulates POMC‐derived neuropeptide release [29] and determines plasticity in PVN MC4R‐expressing neurons [30, 31, 32]. We addressed this question using POMC‐*CB_1_*‐KO mice, which constitutively lack CB1R (gene *Cnr1*) in more than 80% of hypothalamic POMC neurons [12, 13].

First, the lack of CB1R in POMC neurons did not alter body weight (Figure S4A), food intake (Figure S4B), or the refeeding response after a 24 h fast (Figure S4C). Because CB1R signaling in the hippocampus and amygdala critically contributes to the memory processes underlying fear‐related responses [33, 34], we examined whether POMC‐*CB_1_*‐KO mice showed altered CB1R expression in extrahypothalamic regions that express POMC during development [35] or adulthood and that participate in stress regulation [36, 37]. CB1R expression was unaltered in the dentate gyrus of the hippocampus (Figure S4D) and the amygdala (Figure S4E). Importantly, in a classic fear conditioning protocol under 24 h fasting conditions, POMC‐*CB_1_*‐KO and their control littermates had similar immobility responses during acquisition (Figure S5A,B) and retrieval (Figure S5A,C), suggesting that learning processes were not altered by CB1R removal from POMC neurons.

The latency to eat before tone exposure was not impacted by genotype or fear conditioning (Figure 4A). Strikingly, however, the tone‐induced increased latency to feed was blunted in conditioned POMC‐*CB_1_*‐KO mice and significantly reduced compared to conditioned POMC‐*CB_1_*‐Ctrl mice (Figure 4B). Accordingly, fear‐conditioned POMC‐*CB_1_*‐KO mice had lower immobility time during tone presentation than conditioned control littermates (Figure 4C). Altogether, these data indicate that CB1R in POMC neurons is required for the full magnitude of the fear‐suppression response and that its deletion favors feeding over immobility. Of note, this mechanism seems relevant in male, but not in female mice (Figure S5D–F).

**FIGURE 4:**
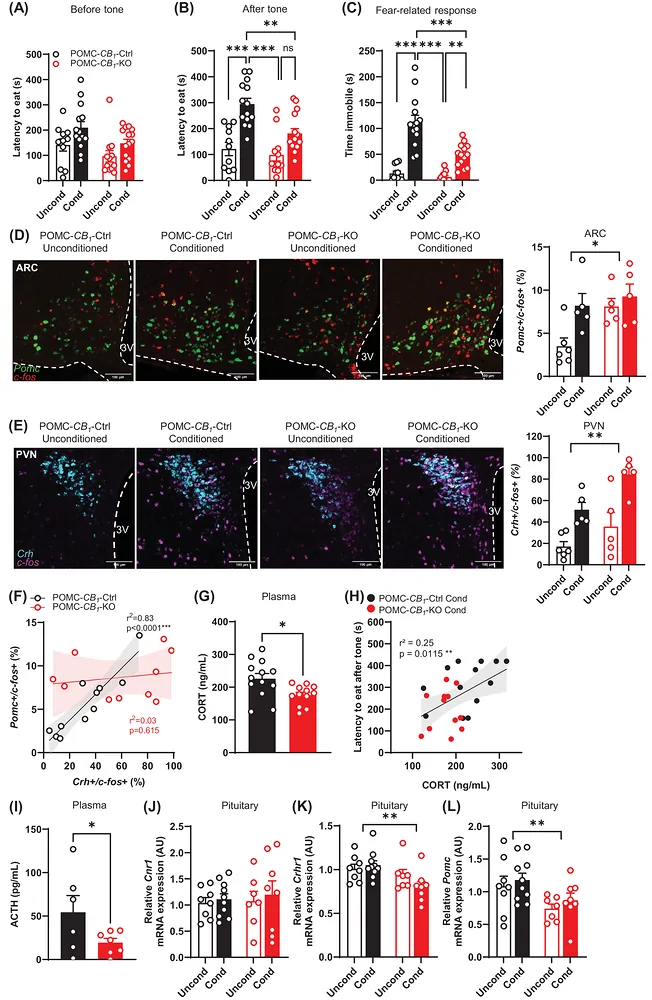
Lack of CB1R in POMC neurons alters prioritization of competing behaviors. Although the latency to eat before the tone was not different between conditioned control and mutant mice (A), latency to eat after the tone was reduced in conditioned POMC‐*CB_1_*‐KO mice (B). (C) Accordingly, conditioned POMC‐*CB_1_*‐KO mice displayed decreased fear‐related response as compared to their conditioned control littermates (*n*  =  11–15 mice/group). (D) Representative images of FISH for *Pomc* and *c‐fos* mRNA expression (*Pomc*, green; *c‐fos*, red) showing increased *c‐fos* expression in *Pomc* neurons of POMC‐*CB_1_*‐KO mice (*n*  =  5–6 mice/group). Genotype effect shown. Scale bars, 100 µm. (E) Representative images of FISH for *Crh* and *c‐fos* mRNA expression in POMC‐*CB_1_*‐KO male mice (*Crh*, turquoise; *c‐fos*, magenta). Mutant mice have increased *c‐fos* expression in CRH neurons (*n*  =  5–6 mice/group). Genotype effect shown. Scale bars, 100 µm. (F) CRH and POMC neuron activation positively correlate in control littermates; however, this correlation is disrupted in mutant mice (*n*  =  10–12 mice/group). (G) Conditioned POMC‐*CB1*‐KO mice have reduced corticosterone plasma levels after the test (*n*  =  12–13 mice/group), and those levels correlate with the latency to eat (*n*  =  12–14 mice/group) (H). (I) Conditioned POMC‐*CB_1_*‐KO mice have decreased ACTH plasma (*n*  =  6–7 mice/group). In the pituitary, POMC‐*CB_1_*‐KO mice had no alterations in *Cnr1* expression (J) but had reduced mRNA expression of *Crh* receptor 1 (*Crhr1*) (K) and *Pomc* (L) (*n* = 15–19 mice/group). Genotype effect shown. Unpaired two‐tailed Student's *t*‐test was performed in (G, I); two‐way ANOVA was applied to (B–E, J–L), followed by Šidák post‐hoc analysis in (B, C); two‐tailed Pearson correlation was used in (F, H). Data are represented as the mean ± s.e.m. ^*^ *p* ≤ 0.05, ^**^ *p* ≤ 0.01, ^***^ *p* ≤ 0.001. ARC: arcuate nucleus of the hypothalamus, PVN: paraventricular nucleus of the hypothalamus, 3V: third ventricle.

### 2.8 CB1R in POMC Neurons Regulate CRH‐HPA Axis Activation

We finally went on to analyze the role of CB1R signaling in POMC neurons on POMC neurons’ activation, and associated changes in CRH neurons and the HPA axis in the context of the fear‐suppression of feeding paradigm. As in C57BL/6N mice (Figure 1I), fear conditioning increased the number of *c‐fos*‐positive *Pomc‐*expressing neurons in conditioned POMC‐*CB_1_*‐Ctrl mice (Figure 4D). This effect, however, was blunted in POMC‐*CB_1_*‐KO mice, since unconditioned and conditioned KO mice displayed a similar number of *c‐fos*‐positive *Pomc‐*expressing neurons (Figure 4D), with no differences in the number of *Pomc*‐expressing neurons (Figure S5G). A genotype effect was also observed for *c‐fos* expression in CRH neurons in the PVN, with mutant mice showing heightened *c‐fos* expression as compared to control littermates (Figure 4E). Again, there were no changes between genotypes in the total number of *Crh*‐expressing neurons (Figure S5H). Interestingly, while in POMC‐*CB_1_*‐Ctrl mice *c‐fos* expression in POMC neurons directly correlated with *c‐fos* expression in CRH neurons, as also observed in C57BL/6N mice (Figure 3B), this correlation was disrupted in POMC‐*CB_1_*‐KO mice (Figure 4F). Altogether, these data reinstate the coordinating role of POMC neurons in suppressing feeding in the presence of a threat, while uncovering the relevance of CB1R signaling in regulating POMC neuron activation and consequent recruitment of CRH neurons in the PVN.

Analysis of the HPA axis further revealed that, in response to the behavioral test, conditioned POMC‐*CB_1_*‐KO mice had significantly decreased CORT plasma levels (Figure 4G) compared to conditioned control littermates. CORT levels directly correlated with the latency to eat after the tone (Figure 4H), linking HPA axis activation to behavioral responses. To then verify the origin of the heightened *c‐fos* expression in POMC and CRH neurons of POMC‐*CB_1_*‐KO mice, which was paradoxically coupled with reduced CORT levels upon conditioning, we investigated more closely the pituitary gland. Similarly to CORT, the pituitary adrenocorticotropic hormone (ACTH) was significantly reduced in the plasma of fear‐conditioned POMC‐*CB_1_*‐KO mice as compared to conditioned control littermates (Figure 4I). Further molecular analysis of the pituitary gland from unconditioned and conditioned mice revealed that no changes in CB1R expression were observed between POMC‐*CB_1_*‐KO and control littermates (Figure 4J). Nevertheless, mutant mice displayed lower mRNA expression of CRH receptor 1 (*Crhr1*) and *Pomc* than controls (Figure 4K,L). The differences between mutant and control mice, however, seemed to be limited to fear stimulation responses, as no genotype effect was observed in basal HPA axis activity. Non‐conditioned POMC‐*CB_1_*‐KO and control littermates displayed similar nictemeral plasma CORT levels, both at the light‐phase nadir and at the dark‐phase onset peak of CORT release (Figure S5I), and performed similarly to controls in behavioral tests that are affected by changes in HPA axis function [4, 36], such as the novelty‐suppression of feeding (Figure S5J,K), the light‐dark box (Figure S5L,M) and the pain sensitivity test (Figure S5N). Thus, these findings imply specific recruitment of POMC neurons and CB1R signaling only when fasted animals face a perceived threat competing with access to food. They also suggest that decreased CORT levels, as a direct marker of a reduced stress‐specific response in the absence of CB1R in POMC cells, may favor feeding over fear in a threatening context, confirming the critical role of POMC neurons and involvement of CRH neurons and the HPA axis in the modulation of behavioral trade‐offs for survival.

## 3 Discussion

Hypothalamic circuits are key to integrating inner states and contextual inputs to balance competing behaviors and ultimately ensure survival according to the energy needs of the organism [3, 4, 5, 7]. In the present study, we investigated the role of hypothalamic POMC neurons during a brief behavioral conflict in which hungry animals must resume feeding despite the retrieval of a learned threat. We identify these neurons as critical regulators of the balance between feeding and defensive behavior. This is likely a coordinated action alongside with other neuronal populations such as the AgRP one, which antagonizes melanocortin signaling [3, 38, 39].

We further found that food‐seeking in the context of threat was associated with increased activation of CRH neurons in the PVN, enhanced HPA axis activity, and elevated circulating CORT levels. Hypothalamic POMC neurons directly project to PVN CRH neurons, and chemogenetic activation of POMC neurons has been shown to increase circulating ACTH and CORT levels [14, 17]. In our paradigm, the correlation between POMC‐ and CRH‐ neuron activation suggests their coordinated recruitment during behavioral adaptation to a threatening context. Although previous work has established a functional interaction between POMC and CRH neurons [14], dedicated circuit‐level experiments will be required to determine whether direct POMC‐to‐CRH connectivity mediates threat‐induced suppression of feeding.

In addition, our data also raise the possibility that the coordinated recruitment of POMC and CRH neurons involves α‐MSH signaling. Increased α‐MSH immunoreactivity in the PVN, together with previous evidence that α‐MSH and MC4R signaling can stimulate CRH release and HPA axis activation [26], supports the biological plausibility of this mechanism. However, α‐MSH immunoreactivity is a static measurement, and future experiments should assess α‐MSH release from POMC neurons, MC4R engagement, or causal activation of PVN CRH neurons. In turn, CORT helps rapidly shift an animal's behavior between competing strategies (e.g., foraging vs. freezing), so that moment‑to‑moment choices favor survival [40]. These rapid, CORT‐dependent effects are exerted through interconnected corticolimbic and hypothalamic circuits [36, 41].

A body of evidence supports the temporal plausibility of the proposed mechanism involving POMC neurons, CRH neurons, and HPA responses in relation to the early (few minutes) behavioral differences we observed in our paradigm. Changes in the activity of POMC and CRH neurons in response to environmental cues and stress are known to occur extremely rapidly, within seconds [9, 14, 16], and engagement of the HPA axis is similarly fast, with CRH transcriptional activation detectable within minutes of stress onset [42]. Consistent with this rapid timescale, our own previous work showed a significant increase in plasma CORT immediately after footshock, demonstrating that circulating CORT can rise within the first minutes following an acute aversive stimulus [43]. Similarly, plasma ACTH has been shown to increase as early as 2 min after the onset of restraint stress [44]. The analysis of rapid behavioral changes and associated neuronal activation was therefore aligned with the test's primary endpoint: capturing the transition from defensive behavior to feeding by measuring the latency to initiate feeding under perceived threat, rather than cumulative food intake. Thus, our findings support a role for POMC neurons in the acute control of threat‐modulated feeding decisions, but do not establish whether modulation of their activity causes sustained changes in food intake or body weight. Longitudinal manipulations will be needed to assess effects beyond the period of learned‐threat retrieval.

Finally, our study reveals that CB1R signaling in POMC neurons is a critical molecular underpinning regulating POMC and CRH neuronal activity as well as the hormonal stress response and consequently behavioral flexibility in the presence of competing demands. Previous studies have shown that CB1R signaling in the PVN inhibits the recruitment of the HPA axis [31, 45]. Here we demonstrate that CB1R in POMC neurons modulates the shift between defensive behavior and feeding and the activity of the HPA axis in a context‐dependent manner. Specifically, CB1R in POMC neurons is necessary for the normal magnitude of the fear‐suppression of feeding response. Despite the learnt threat, mice lacking CB1R in POMC neurons approach food more rapidly than control animals, likely because of a blunted release of ACTH and CORT in this specific context.

Notwithstanding, genetic deletion of CB1R in POMC neurons results in sustained hyperactivation of POMC cells. In line with this finding, our previous studies have shown that genetic removal of CB1R can increase c‐Fos expression in the very neurons that lack CB1R (such as excitatory neurons in the medial habenula) [46], indicating that basal and/or evoked activity is directly changed. POMC neuron overactivation in POMC‐*CB_1_*‐KO mice is accompanied by persistently increased *c‐Fos* expression in PVN CRH neurons. Although α‐MSH was not investigated, CB1R deletion in POMC neurons may alter α‐MSH signaling onto CRH neurons. Based on previous evidence, this could occur through several, potentially complementary mechanisms: (i) CB1R‐dependent changes in POMC neuronal excitability [45]; (ii) an effect of CB1R signaling on the preferential release of specific POMC‐derived peptides [29]; and/or (iii) altered postsynaptic melanocortin signaling in downstream PVN neurons [12, 13].

Whilst the reduced mRNA expression of *Crhr1* and *Pomc* at the level of the pituitary gland of POMC‐*CB_1_*‐KO mice may reflect a compensatory adaptation inherent to this constitutive genetic model. Although this adaptation does not appear to affect basal circadian CORT rhythmicity, it could compromise hormonal responsiveness under a threat situation. The mechanisms underlying this potential impairment remain unclear and warrant further investigation. Alternatively, or in parallel with an altered pituitary response, diminished glucocorticoid‐mediated negative feedback to the hypothalamus could further contribute to the sustained overactivation of POMC and CRH neurons observed in POMC‐*CB_1_*‐KO mice.

## 4 Conclusion

Collectively, these findings identify a previously underappreciated role for hypothalamic POMC neurons in survival‐related decision‐making. Our data support the coordinated recruitment of POMC and PVN CRH neurons, together with HPA‐axis activation when metabolic need conflicts with a learned threat. CB1R signaling in POMC neurons emerges as a key molecular mechanism underlying this behavioral flexibility, as its deletion attenuates fear‐induced suppression of feeding and alters neuronal and endocrine stress responses. Although these findings provide a mechanistic framework, future work using temporally precise, cell‐type‐ and projection‐specific approaches will be required to establish the causal POMC‐to‐CRH circuit mechanisms involved. Given the profound influence of stress and environmental factors on metabolic homeostasis, mood, and behavior, this work may help clarify how dysregulated stress responses and maladaptive threat evaluation contribute to metabolic and psychiatric disorders.

## 5 Methods

### 5.1 Animals

All procedures involving live animals were approved and conducted in strict compliance with the National and European Union Directives (2010/63/EU), the French Ministry of Agriculture, Agri‐Food and Food Sovereignty, and the Ethical Committee of the University of Bordeaux for Animal Experimentation (NTS‐FR‐283735). Procedures followed the ARRIVE guidelines. C57BL/6N mice (Janvier, France), POMC^Cre^ mice (Tg(Pomc1‐cre)16Lowl/J, #JAX:005965, The Jackson Laboratory, USA), POMC‐*CB_1_*‐KO, POMC^CreERT2^; vGlut2^fl/fl^ (thereafter called POMC‐*vGlut2*‐KO after tamoxifen administration), and their control littermates were used. POMC‐*CB_1_*‐KO were generated as previously described [12, 13] by crossing POMC^Cre^ mice with CB1^fl/fl^ mice [47]. Control littermates of POMC‐*CB_1_*‐KO mouse line were used for the Figure S1B,C and for the Figure S2C–G. Tamoxifen‐inducible POMC‐*vGlut2*‐KO were generated by crossing POMC^CreERT2^mice (C57BL/6J‐Tg(Pomc‐creERT2)) [48], a kind gift of Dr. Joel Elmquist at UT Southwestern, with vGlut2^fl/fl^ mice (Slc17a6tm1Lowl/J, #JAX:012898). POMC^Cre‐ERT2^*Ai6 reporter mouse (Gt(ROSA)26Sortm6(CAG‐ZsGreen1)Hze, # JAX:007906) was used to test the expression of Cre after tamoxifen treatment. All mouse lines were generated in the animal facility of the Neurocentre Magendie. All animals were housed under a 12:12 h reversed light/dark cycle (lights on at 3:00 h), at 22 ± 2°C, with ad libitum access to water and food (Standard Rodent Diet A03, SAFE, France). Mice were kept in collective cages until the behavioral tests were carried out between 9 and 12 weeks of age. One week before the experiments started, mice were housed in individual cages. Both collective and individual cages were always enriched with bedding, a cardboard tunnel, a wooden stick, and with a red plastic house. The number of animals used in each experiment is indicated in figure legends.

### 5.2 AAV Vectors

AAV‐8/2‐DIO‐hM4D(Gi)‐mCherry (7.7 × 10^12^ vg mL^−1^, Viral Vector Facility, University of Zurich, ETH Zurich, Neuroscience Center Zurich, Switzerland) was injected into the ARC (see below) of POMC^Cre^ mice for chemogenetic inhibition.

### 5.3 Stereotaxic Surgery

Mice were anaesthetized using 5% isoflurane and placed into the stereotaxic apparatus (David Kopf Instruments) with a mouse adaptor and lateral head bars, and the isoflurane was maintained at 1.5%–% during the surgery. Subcutaneous buprenorphine (0.1 mg kg^−1^, Bupaq, Vibrac) and lidocaine (0.1 mL at 0.5%, Lidor, Centravet) at the level of the skull were injected prior to surgery to induce analgesia. A heating pad was positioned underneath the animal to avoid hypothermia. Eye ointment was applied to keep the eyes from drying (Ocrygel Carbomere, Centravet). The skin above the skull was shaved with an electric razor and disinfected with iodine solution (Betadine) before an incision was made. Viral vectors were injected with a glass pipette attached to a microinjector (Nanoject III, Drummond Scientific). AAV vector (250 nL) was bilaterally infused into the ARC (coordinate AP/DV/ML = −1.5/−5.9/±0.3 mm; infusion speed = 2 nL s^−1^) of POMC^Cre^ mice. The same procedures were used also in control Cre^−^ mice. Following virus delivery, the glass pipette was held in place for 5 min after injection to avoid viral diffusion. All mice received saline solution (0.3 mL) subcutaneously, and meloxicam (5 mg kg^−1^, Metacam, Centravet) for 2 days. Animals continued to be housed collectively, and body weight was monitored daily for 1 week to assess recovery. Behavioral experiments were carried out at least 4 weeks after surgery.

### 5.4 Tamoxifen Treatment for the Induction of Cre‐Mediated Recombination

For POMC‐*vGlut2*‐KO, corn oil (Sigma–Aldrich, France), pre‐heated to 55°C, was added to the suspension of tamoxifen (1 g mL^−1^, Sigma–Aldrich, France) in 100% ethanol (VWR, France), obtaining a tamoxifen stock solution (50 mg mL^−1^). Mice received tamoxifen (150 mg kg^−1^) daily for 5 consecutive days by oral gavage (3 mL kg^−1^) done using polypropylene flexible gavage needles (Instechlabs, Germany).

### 5.5 Behavioral Tests

All behavioral experiments were performed during the light phase, with the light intensity monitored and low noise conditions. On the day of the experiment, mice were habituated to the room for 1 h before the experiment. For the phenotypic characterization of POMC‐*CB_1_* mice, when the same cohort was used for multiple tests, the most stressful assays were performed last, to minimize between‐test interference.

#### 5.5.1 Fear‐Suppression of Feeding

This paradigm is an adaptation of classical auditory fear‐conditioning, allowing to study fear‐related response and eating behavior simultaneously [15], and it was performed in C57BL/6N, POMC^Cre^, POMC‐*CB_1,_* and POMC‐*vGlut2* mice. On day 1, mice were trained in a fear conditioning apparatus in context A (Plexiglas cage with dark grey walls and metal grid floor, see Figure 1A). Fear conditioning was performed by placing the mice in context A for 2 min acclimation period, followed by three conditional–unconditional stimuli (CS‐US) separated by 1 min interval. The CS‐US consisted of 3 tones (CS, 20 s, 65 dB) co‐terminating with a foot shock (US, 0.5 mA, 2 s, constant current). Half of the mice didn't receive the shock (“Unconditioned” group), while the other half did (“Conditioned” group). The fear conditioning chamber was thoroughly cleaned with Phagospray (Laboratoire Phagogène, France) before each mouse was placed in the box. After training, mice were returned to their home cage for 30 min and then transferred to a new clean cage for 24 h without food but preserving their bedding, tunnel, and wood stick. After fasting, on the second day, mice were transferred to a novel environment (Plexiglas cage with transparent walls with orange patterns and a Plexiglas white floor, see Figure 1A), with a chow food pellet placed on the floor, in a corner. Mice were allowed to explore the box until the first bite of the food pellet, with the light on and without the tone. If mice were taking longer than 10 min to eat, they were excluded from the experiment. Immediately after mice started to eat, the tone was played for 7 min consecutively. The latency to eat before and after the tone, the total food consumption, as well as the immobility time were measured manually. For studies that required brain sample collection for immunofluorescence or fluorescent in situ hybridization, or blood collection for corticosterone and ACTH measurements, the test was stopped right after the latency to eat after the tone, with the objective of assessing physiological changes associated with the latency‐to‐eat period while minimizing the contribution of subsequent food consumption. For chemogenetic studies, Clozapine‐N‐oxide (CNO, Hello Bio, Republic of Ireland) was diluted in saline (5 mg kg^−1^), and mice were injected subcutaneously 1 h before the memory retrieval test on day 2. For the fear‐conditioning test without food, the protocol was the same, but on day 2 the food pellet was not presented, and the test was stopped after the 7 min tone (Figure S5A).

#### 5.5.2 Feeding Behavior and Body Weight Assessment

Food intake and body weight were measured for 7 consecutive days in male 8‐weeks‐old POMC‐*CB_1_* mice. At 10‐weeks‐old, mice were fasted for 24 h, and the food consumption and body weight were monitored for 2 h after access to food.

#### 5.5.3 Novelty‐Suppression of Feeding

The test was performed in a white open‐field arena (60 × 60 cm, light intensity at the center 50 lux) with a food pellet position in the center of the maze. POMC‐CB1 mice were fasted for 24 h before the experiment and recorded in the maze for 5 min. The feeding behavior was assessed manually by measuring the total food consumed and the latency to eat.

#### 5.5.4 Light‐Dark Box

It consisted of a black Plexiglas compartment (20 × 30 cm) and an illuminated compartment (30 × 30 cm) communicating through an opening. The light intensity was set up at 300 lux in the middle of the light compartment and 15 lux in the middle of the dark compartment. POMC‐*CB_1_* mice were positioned in the dark compartment facing the opening and allowed to move freely for 5 min. The latency to the light compartment, defined by entering with all four paws into the compartment, as well as the time spent in the light compartment, was measured manually.

#### 5.5.5 Hot Plate

POMC‐*CB_1_* mice were placed individually on a hot plate with the temperature adjusted to 52°C. The latency of the response to jump or lick the hind paws was measured. The cut‐off time was of 30 s, to avoid risk of thermal injury to the skin.

### 5.6 Tissue Sample Collection

Mice were deeply anesthetized using with intraperitoneal solution (10 µl g^−1^) consisting of pentobarbital (300 mg kg^−1^, Euthasol, Centravet) and Lidocaine (30 mg kg^−1^, Lidor, Centravet) and then perfused transcardially with ice‐cold home‐made phosphate buffered saline (PBS), pH 7.4, for 30 s followed by 4% paraformaldehyde (PFA) / 10% neutral buffered formalin (NBF, Merck, #HT501320) in PBS for 2 min at a constant rate (7.5 mL min^−1^). Brains were extracted and postfixed in 4% PFA overnight at 4°C, then cryoprotected with 30% sucrose in PBS at 4°C for a minimum of 72 h. Coronal sections (30 µm) were cut with a cryostat (CM1950, Leica, Germany), collected and stored in antifreeze solution (30% ethylene glycol, 20% glycerol in PB) at −20°C until further use. For c‐Fos and POMC protein detection, animals were perfused 90 min after the start of the behavioral test, whereas for *c‐fos*, *Crh*, and *Pomc* mRNA, as well as α‐MSH protein, perfusion occurred at 15 min after test onset.

Blood samples were collected from the tail for a maximum of 2 min in capillaries prepared with K3 Ethylenediaminetetraacetic acid (EDTA) for measurement of corticosterone in the light and at the onset of the dark phase. Trunk blood was collected in EDTA tubes 30 min after the start of the fear‐suppression of feeding test for corticosterone measurement, or 15 min after the start of the test for ACTH measurement. Plasma was obtained by centrifugation step at 10000 g for 10 min using a microcentrifuge. Plasma was collected and kept at −80°C until used. Mice were sacrificed by dislocation, and pituitary samples were rapidly collected, weighed, frozen in liquid nitrogen, and kept at −80°C until further use.

### 5.7 Immunofluorescence

For the detection of c‐Fos protein in POMC neurons, mice completed the full 7‐min fear‐suppression‐of‐feeding test and were perfused 90 min after the beginning of the behavioral test. Four brain sections from each mouse containing the hypothalamus were used. Sections were washed three times for 5 min with PBS (0.01 m). Afterward, samples were incubated for 30 min on an agitator with blocking solution containing PBS (0.01 m) + 0.03% Triton, Glycine (50 mm), and 2% Normal Donkey Serum (NDS) to block antibody binding to non‐specific proteins. Samples were then incubated overnight at room temperature with sheep anti‐αMSH (1:5000) (Millipore, # AB5087), diluted in PBS (0.01 m) + 0.3% Triton, Glycine (10 mm), and 0.1% H_2_O_2_. The following day, brain sections were incubated with donkey anti‐goat‐647 (1:500) (Invitrogen, # A21447) in incubation buffer (0.01 m PBS + 0.03% Triton) for 2 h in the dark. We then incubated the slices in DAPI (0.25 µg mL^−1^) for 5 min, then washed three times for 5 min in Tris‐HCl (50 mm, pH = 7.5). The sections were then mounted onto gelatin‐coated glass slides and coverslipped, using ProLong Gold (Invitrogen, # P36930) as the mounting medium.

For the staining of POMC and c‐Fos, after the first three washes with 0.01 m PBS, samples were incubated in blocking solution as described above. Subsequently, slices were incubated overnight at room temperature with primary antibodies rabbit anti‐POMC (1:2000) (Phoenix Pharmaceuticals, #H‐029‐30) and guinea‐pig anti‐c‐Fos (1:1000) (Synaptic Systems, #226005), diluted in PBS (0.01 m) + 0.3% Triton, Glycine (10 mm), and 0.1% H_2_O_2_. The following day, each well was washed three times with PBS (0.01 m) and incubated in the dark for 2 h with secondary antibodies donkey α‐rabbit‐647 (1:500) (Jackson ImmunoReasearch, # 711‐605‐152) and donkey α‐guinea‐pig‐488 (1:500) (Jackson ImmunoReasearch, # 706‐545‐148) diluted in PBS (0.01 m) + 0.3% Triton. As described above, samples were washed, then incubated with DAPI and mounted with ProLong Gold antifade reagent.

For the staining of POMC in DREADD‐expressing samples, sections were washed and blocked as described above. Then, slices were incubated overnight at room temperature with primary antibody rabbit anti‐POMC (1:2000) (Phoenix Pharmaceuticals, #H‐029‐30), diluted in PBS (0.01 m) + 0.3% Triton and Glycine (10 mm). H_2_O_2_ was omitted to preserve endogenous mCherry fluorescence. The following day, after three 5‐min washes with PBS (0.01 m), sections were incubated in the dark for 2 h with secondary antibody donkey α‐rabbit‐647 (1:500) (Jackson ImmunoResearch, # 711‐605‐152), diluted in PBS (0.01 m) + 0.3% Triton. Sections were then incubated in DAPI, washed, and coverslipped as described above.

### 5.8 Double Fluorescent In Situ Hybridization (FISH)

Digoxigenin‐labeled riboprobe against mouse *c‐fos‐*DIG (Allen Brain Atlas, # RP_080109_01_B10), fluorescein‐labeled riboprobe against mouse *Pomc* (*Pomc‐*FITC, Allen Brain Atlas, #RP_Baylor_102974), *Crh‐*FITC (*Crh‐*FITC, Allen Brain Atlas, # RP_Baylor_102704) and *Cnr1‐DIG* [49] were prepared from hypothalamic RNA reverse transcribed into cDNA as described elsewhere [50]. DNA templates for *c‐fos*, *Pomc*, *Crh* and *Cnr1* were obtained via PCR and sub‐cloned into pBluescript vector using the restriction enzymes EcoRI and BamHI for *Pomc, c‐fos and Cnr1*, and XbaI and EcoRI for *Crh*. After linearization with BamHI for *Pomc, c‐fos*, *and Cnr1*, and XbaI for *Crh*, the antisense riboprobes were synthesized with T3 RNA polymerase (Roche, # 11031163001). For the generation of sense riboprobes, EcoRI (*Pomc*, *c‐fos*, *Crh*, and *Cnr1*) was used for linearization and T7 RNA polymerase (Roche, # 10881767001) for the synthesis. The riboprobes were produced by in vitro synthesis, using 10 X DIG RNA labeling mix (for *c‐fos*‐DIG and *Cnr1*‐DIG, Roche, # 11277073910) or 10 X FITC RNA labeling mix (for *Pomc*‐FITC and *Crh*‐FITC, Roche, # 11685619910). The riboprobes were purified using the Monarch Spin RNA cleanup kit as per the manufacturer's instructions (New England Biolabs, # T2030).

For each riboprobe, the concentration was measured using a NanoDrop and adjusted at 200 µm by addition of the required volume of hybridization buffer (see below). FISH was performed as follows. Free‐floating sections were treated with HCl (0.2 m) for 20 min, then acetylated with 0.25% acetic anhydride in triethanolamine (0.1 m, pH = 8.0) for 10 min, before hybridization. Between all steps, sections were rinsed for 5 min in PBS treated beforehand with 0.1% diethylpyrocarbonate (DEPC), then autoclaved. *Pomc*‐FITC with *c‐fos*‐DIG, *Crh*‐FITC with *c‐fos*‐Dig or *Cnr1*‐DIG alone were diluted 1:1000 in hybridization buffer containing 50% deionized formamide, Tris (20 mm, pH = 8.0), NaCl (300 mm), EDTA (5 mm), 10% dextran sulfate, 1X Denhardt's solution, tRNA (0.5 mg mL^−1^), acid‐cleaved carrier DNA from salmon's sperm (0.2 mg mL^−1^) and DTT (200 mm) dissolved 0.1% DEPC autoclaved water, heated at 90°C for 5 min and incubated overnight at 70°C. After hybridization, sections were washed at 70°C with saline‐sodium citrate buffer of increased stringency (5X SSC for 5 min, 2X SSC + 50% Formamide, 1X SSC + 50% Formamide and 0.1X SSC for 30 min each, with 0.2% Tween 20 added to each buffer). After blocking 30 min in TNB blocking buffer supplemented with 4% sheep serum (AkoyaBiosciences, # FP1020), sections were incubated 1 h at room temperature with a sheep anti‐DIG‐POD antibody (1:2000, Roche, # 11207733910) in TNB. The revelation was made using a TSA Plus Cyanine 3 kit (1:100, 10 min, Akoya Biosciences, # NEL744001KT). After quenching with 3% H_2_O_2_ in PBS (30 min) and HCl (0.2 m) for 20 min, sections were incubated in sheep anti‐FITC‐POD antibody (1:2000, Roche, # 11426346910) in TNB overnight at 4°C and the revelation made using TSA Plus Fluorescein kit (1:100, 10 min, AkoyaBiosciences, # NEL741001KT).

### 5.9 Image Acquisition and Data Analysis

Pictures of *Pomc*, *Crh, c‐fos*, and *Cnr1* FISH staining and immunofluorescence for POMC and c‐Fos staining were captured using a confocal microscope with objective 20X dry (SP8‐STED, Leica) at a resolution of 2048 × 2048, 12 bits, and fixed acquisition parameters. The sampling rate respected the optical resolution of the system (voxel size: 0.0819 × 0.0819 × 0.2985 µm^3^) and *z* axis stacks were processed (10 stacks per image). Images were acquired separately for each wavelength (blue = 405 nm, green = 488 nm, red = 552 nm, and far red = 638 nm) and then merged for quantification and representation.

For quantification, all channels were left unchanged, and an average of 2 hemi‐ARC sections were quantified per mouse from the same rostro‐caudal level. Maximum intensities were projected using the ImageJ software (National Institutes of Health, USA) and then analyzed with the open software QuPath [51]. The region of interest (ROI) was determined in each picture according to *Pomc* or *Crh* staining, and the positive cells were detected using the Positive Cell Detection plugin. *Pomc* or *Crh* positive cells were detected, and an intensity threshold was set for *c‐fos*, obtaining the annotations datasheet with the positive and negative cells for *c‐fos* and the detection data, obtaining the intensity of *Pomc*, *Crh*, and *c‐fos* per cell. *Cnr1* positive neurons in the hippocampus and amygdala were quantified using the Cell Detection plugin from QuPath, and the ROIs were recognized according to the Allen mouse brain atlas.

Immunofluorescence staining of α‐MSH as well as of POMC in DREADD‐expressing samples was captured with a Leica fluorescence microscope (DM 4000 B LED), equipped with a digital monochrome camera (DFC 365 FX). The signal of α‐MSH was quantified using the ImageJ software. The PVN area was determined using DAPI staining according to the Allen mouse brain atlas from the same rostro‐caudal level and the ROI intensity was measured and normalized by the ROI intensity of non‐positive non‐PVN area (background).

For illustration purposes of FISH and immunofluorescence, images were subjected to brightness and contrast enhancement that were kept constant for each channel across all related conditions.

### 5.10 Measurement of Plasma Corticosterone and ACTH Levels

Corticosterone levels were measured by performing enzyme‐linked immunosorbent assay (ELISA) analysis following the manufacturer's instructions (DetectX Corticosterone Immunoassay Kit; Arbor Assays, # K014‐H). In brief, samples were mixed 1:1 with Dissociation Reagent and incubated at room temperature (RT) for 5 min. All samples were then diluted in Assay Buffer at a ratio of 1:100. Then DetectX Corticosterone Conjugate (25 µl) and DetectX Corticosterone Antibody (25 µl) were added, and the plate was incubated at RT for 1 h while shaking at 700–900 rpm. Wells were washed four times with wash buffer (300 µL), 100 µL of the TMB Substrate was added to each well and the plate was incubated at RT for 30 min without shaking. After adding 50 µL of Stop Solution to each well, the optical density generated in each well was immediately read at 450 nm using the POLARstar Omega plate reader (BMG Labtech, Germany).

ACTH levels were measured using an ELISA kit (Enzo Life Sciences, Switzerland, ENZ‐KIT138) following the manufacturer's recommendations with some modifications to adjust it to mice samples. Briefly, 50 µL of plasma per well was used instead of 200 µL, and the volume of the calibrators, controls, and buffers escalated accordingly. Biotinylated Antibody and Enzyme Labeled Antibody and incubated for 4 h at RT. After five washes with Working wash solution, the TMB was added and incubated for 35 min at RT, and the reaction was stopped with the addition of Stop solution. Right after, the absorbance was read at 450 nm using the POLARstar Omega plate reader.

### 5.11 PCR for Genotyping

Mice were genotyped by the genotyping facility of the Neurocentre Magendie, from genomic DNA purified from tail biopsies by PCR using specific primers for Cre and vGlut2‐flox (Table 1). POMC^Cre^ mice and CB1^fl/fl^ mice were genotyped as previously described [13]. For analysis of vGlut2 excision in POMC‐*vGlut2*‐Ctrl/KO mice, fresh hypothalami were dissected on ice and snap frozen after cervical dislocation. Tails or hypothalami were incubated overnight at 56°C in Proteinase K buffer (50 mm Tris‐HCl pH = 8.5, 10 mm EDTA pH = 8, 0.5% SDS, 200 mm NaCl, 0.15 mg mL^−1^ PK). After 10 min at 16300 g, the supernatants were purified on silica columns, according to the manufacturer's protocol (Macherey nagel kit), on a zephyr automatic station. PCR assay was carried out using a Bio‐Rad C1000 thermal cycler, with GoTaq G2 Hot Start Green Master Mix (Promega, Charbonnières‐les‐Bains, France), and 0.3 to 0.6 µm of each primer (see Table 1) in a 12 µL volume. PCR conditions were as follows: 1 cycle, 5 min at 95°C; 37 cycles, 30 s at 95°C, 30 s at 58°C for, 50 s at 72°C; 1 cycle, 5 min at 72°C. PCR products were analyzed on a Labchip GX microfluidic electrophoresis system (Revvity) using the DNA5k kit.

**TABLE 1:** Primer Sequences for PCR.

Mouse line	Primer	Sequence 5′ ‐ 3′	Expected fragment	Length
**POMC‐CreER^T2^**	Cre forward	CGGCATGGTGCAAGTTGAATA	Cre	300pb
	Cre reverse	GCGATCGCTATTTTCCATGAG		
	β‐actin forward	TTCTTTGCAGCTCCTTCGTTGCCG	β‐actin	450pb
	β‐actin reverse	TGGATGGCTACGTACATGGCTGGG		
**vGlut2‐flox**	vGlut2 forward	CTGAGCGAAGGTGAGCTGAA	Flox	400pb
	vGlut2 reverse	TGGGCCAGAACACAGGATATG	WT	270pb
	vGlut2 fwd. excision	TCACTGCCTTGTTCCTAGTGC	Excision	700pb

### 5.12 Quantitative Real Time (qPCR)

qPCR was carried out in pituitary samples of POMC‐*CB_1_*‐KO and their control littermates after the fear‐suppression of feeding for *Pomc*, *Crhr1* and *Cnr1*. Total RNA was extracted by using the Agencourt RNAdvance Tissue (Beckman Coulter, France) according to the manufacturer's protocol and eluted with 40 µl of RNAse‐free water. RNA was processed and analyzed according to an adaptation of published methods [52]. cDNA was synthesized from 1 µg of total RNA by using Maxima Reverse Transcriptase (Thermo Scientific, France). qPCR was performed with a LightCycler 480 Real‐Time PCR System (Roche, Switzerland). qPCR reactions were done for each sample, using transcript‐specific primers, cDNA (4 ng), and LightCycler 480 SYBR Green I Master (Roche, Switzerland) in a final volume of 10 µl. The qPCR data were exported and analyzed in an informatics tool (Gene Expression Analysis Software Environment) developed at the Neurocentre Magendie. For the determination of the reference genes, the RefFinder method was used [53]. Relative expression analysis was normalized against two reference genes: Elongation factor 1‐alpha 1 (*Eef1a1*) and succinate dehydrogenase complex subunit (*Sdha*). The relative level of expression was calculated with the comparative (2^−ΔΔCT^) method [54]. Primer sequences are reported in Table 2.

**TABLE 2:** Primer Sequences for qPCR.

Gene	GenBank ID	Forward Sequence (5′‐3′)	Reverse Sequence (5′‐3′)
Pomc	NM_008895	TGGGCGAGCTGATGACCT	GCCGACTGTGAAATCTGAAAGG
Crhr1	NM_007762	CCTCTGGGTATCTTCTAACCTGTGA	ACTCTGGTTCCTGGCAACTCA
Cnr1	NM_007726	TCAGTTGCTGTGGAATCTTTAAAAA	CTGATCGCAGGACCCCTAGA
Eef1a1	NM_010106	TGAACCATCCAGGCCAAATC	GCATGCTATGTGGGCTGTGT
Sdha	NM_023281	CTGTCTGGTGCATTCCTGAACTAT	TGTTCCCCAAACGGCTTCT

### 5.13 Statistical Analysis

Statistical analyses were performed using Prism 10 (GraphPad Software, La Jolla, CA, USA). Potential outliers were assessed for all datasets using the robust regression and outlier removal method, with the maximum false discovery rate set at Q = 1%. Data distribution was assessed before statistical analysis, and parametric or non‐parametric tests were applied accordingly, as specified in the figure legends. All values, unless stated otherwise in the figure legends, are reported as mean ± s.e.m. Data were analyzed by unpaired or paired Student's *t* tests, one‐way or two‐way ANOVAs, as appropriate. Repeated measures (RM) for matched individuals were used when the same animals were measured at different time points. Significant interaction in ANOVAs was followed by Šidák's post‐hoc test. To describe correlation, linear regression analysis and Pearson correlation test were performed. P<0.05 denotes statistical significance.

## Author Contributions

**D.C., C.M. and A.E.‐P**. conceived and designed the study and wrote the manuscript, with critical contributions from **G.M. and C.Q. C.M., and A.E.‐P**. performed the majority of the experiments and analyzed the data. **V.S., V.J., M.S., S.C., P.Z., U.S., A.C., N.D., J.C.N., A.C., T.L.L. and G.L**. contributed to the experiments and/or data analysis. All authors edited and approved the final manuscript.

## Conflicts of Interest

The authors declare no conflicts of interest.

## Supporting information




**Supporting File**: advs78379‐sup‐0001‐SuppMat.pdf.

## Data Availability

Source data and representative images are provided with this paper. All other data supporting the findings of this study are available from the corresponding authors upon reasonable request.
