# Supplementary material for: Hypothalamic POMC Neurons Gate the Fear‐Feeding Trade‐Off
Source: Adv Sci (Weinh). 2026 Oct 20:e78379. Online ahead of print. doi: 10.1002/advs.78379 (PMC13683886; doi:10.1002/advs.78379)
Supplement: Supplementary file 1 [file ADVS-9999-e78379-s001.pdf]

# Supporting information

**Hypothalamic POMC Neurons Gate the Fear-Feeding Trade-off**

*Cristina Miralpeix*, Abel Eraso-Pichot, Vincent Simon, Victor Jouque, Melissa Sadallah, Samantha Clark, Philippe Zizzari, Urszula Skupio, Ana Covelo, Nathalie Dupuy, Jean-Charles Nicolas, Astrid Cannich, Thierry Leste-Lasserre, Guillaume Laplagne, Carmelo Quarta, Giovanni Marsicano, Daniela Cota*.*

*Corresponding authors emails: cristina.miralpeix.monclus@gmail.com; daniela.cota@inserm.fr

**This file includes:**
Figures S1 to S5

# Figure S1

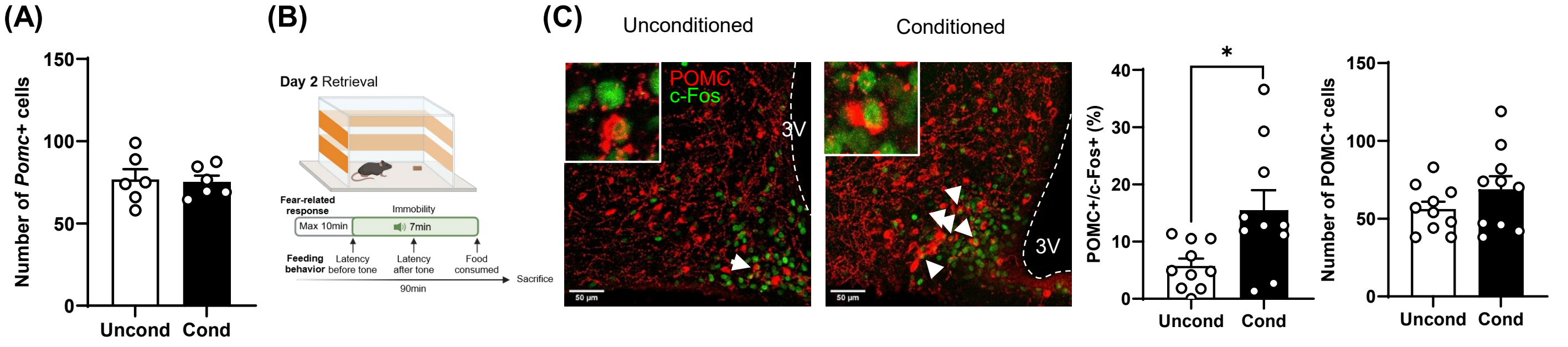


**Figure S1.** (A) Fear suppression of feeding in mice does not modify the mean number of *Pomc*+ cells in the ARC ($n$ = 6 mice/group). (B) For c-Fos protein assessment in POMC neurons, mice underwent the fear-suppression of feeding paradigm and were sacrificed 90 minutes later for tissue collection. (C) Conditioned mice also had increased expression of c-Fos protein in POMC neurons ($n$ = 10 mice/group), with unaltered number of POMC+ neurons. Data are represented as the mean ± s.e.m. *$P \leq 0.05$. Unpaired two-tailed Student's t-test in C.

# Figure S2

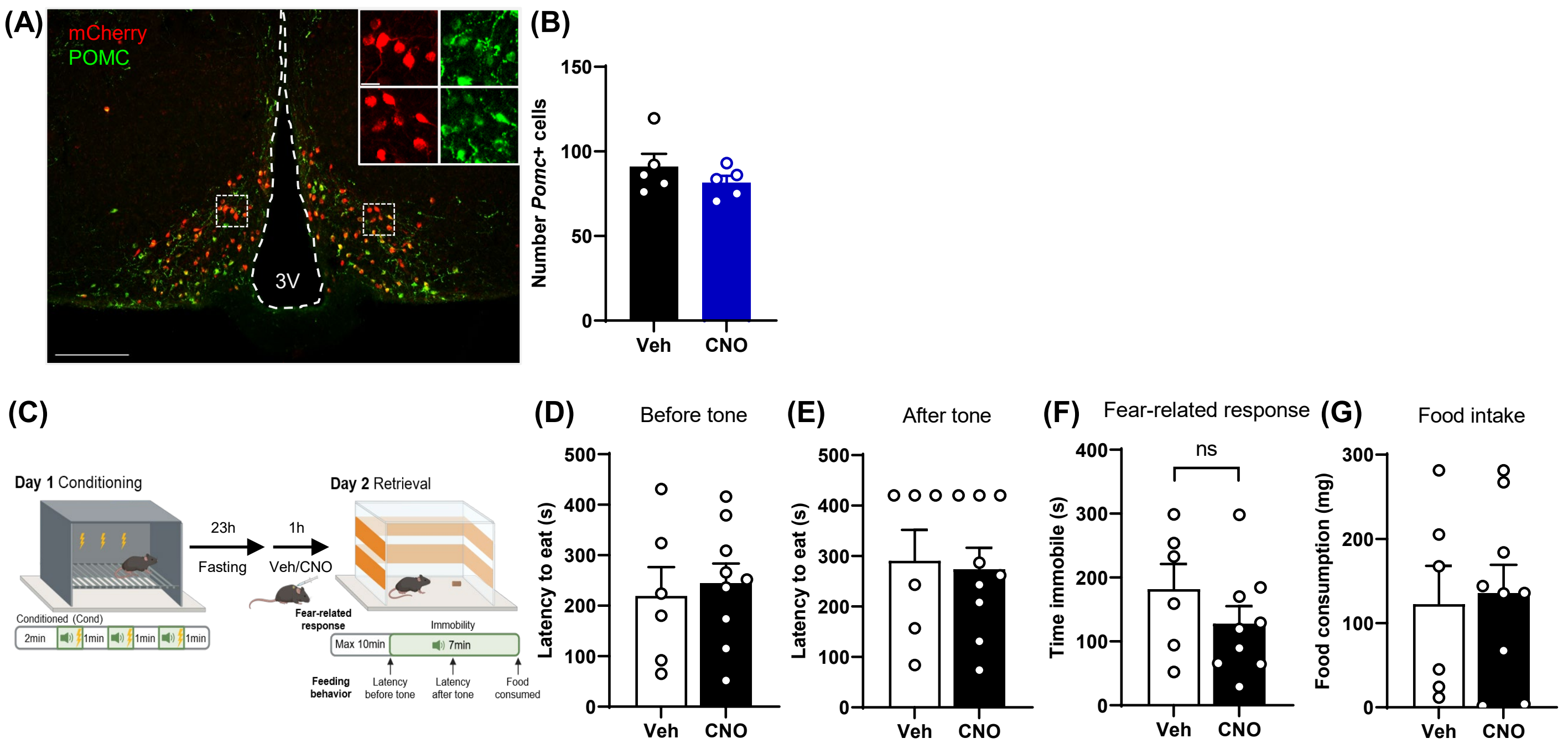


**Figure S2.** (A) Representative image of DREADD hM4Di-mCherry (red) expression in POMC neurons (green) in POMC-Cre mice. Scale bar = 200 µm; scale bar inset = 20 µm. (B) CNO injection did not modify the number of cells expressing *Pomc*+ in the ARC (*n* = 5 mice/group). (C) Control Cre$^{-}$ mice were used to study the potential DREADD-independent effects of 5mg/kg CNO in the fear suppression of feeding test. (D-G) CNO *per se* does not alter the latency to eat before the tone (D), the latency after the tone (E), the fear-related response (F), or the total food consumption (G) during the 7-minute fear-suppression of feeding test (*n* = 6 - 9 mice/group). Data are represented as the mean ± s.e.m.

## Figure S3

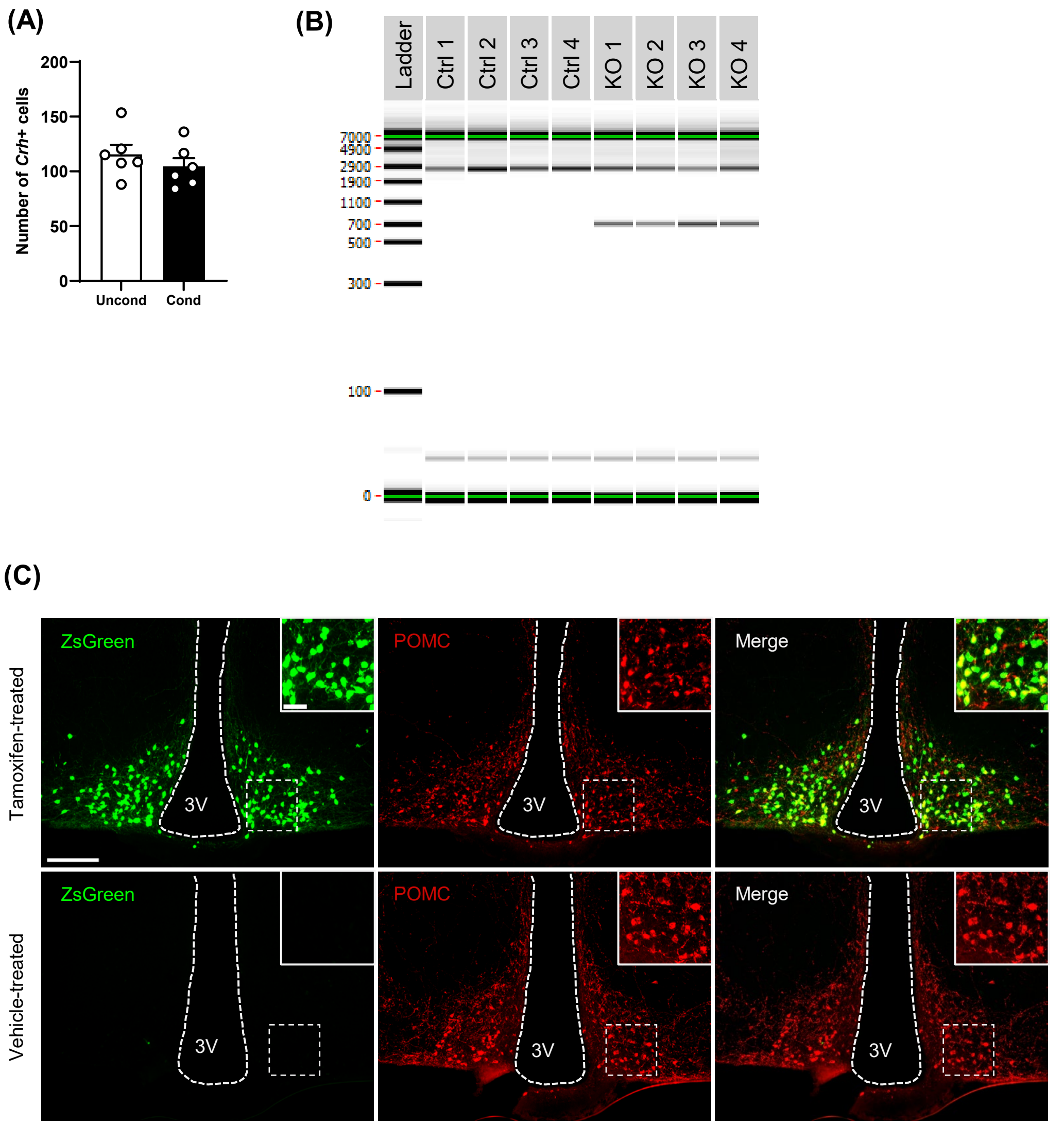


**Figure S3.** (A) Mean number of *Crh+* positive neurons in the PVN did not differ between unconditioned and conditioned groups (*n* = 6 mice/group). (B) Example PCR of hypothalami of control littermates and POMC-*vGlut2*-KO mice after Tamoxifen treatment. KO mice show a double DNA band corresponding to the excision of the *Slc17a6* (vGlut2) gene. (C) Representative images of tamoxifen-induced expression of ZsGreen (green) signal corresponding to Cre expression in POMC neurons (red). Images were obtained from Tamoxifen treated POMC$^{Cre\text{-}ERT2}$*Ai6 reporter mouse. Scale bar low magnification = 200 µm. Scale bar high magnification inset = 50 µm. Data are represented as the mean ± s.e.m. ARC: arcuate nucleus of the hypothalamus, 3V: third ventricle.

# Figure S4

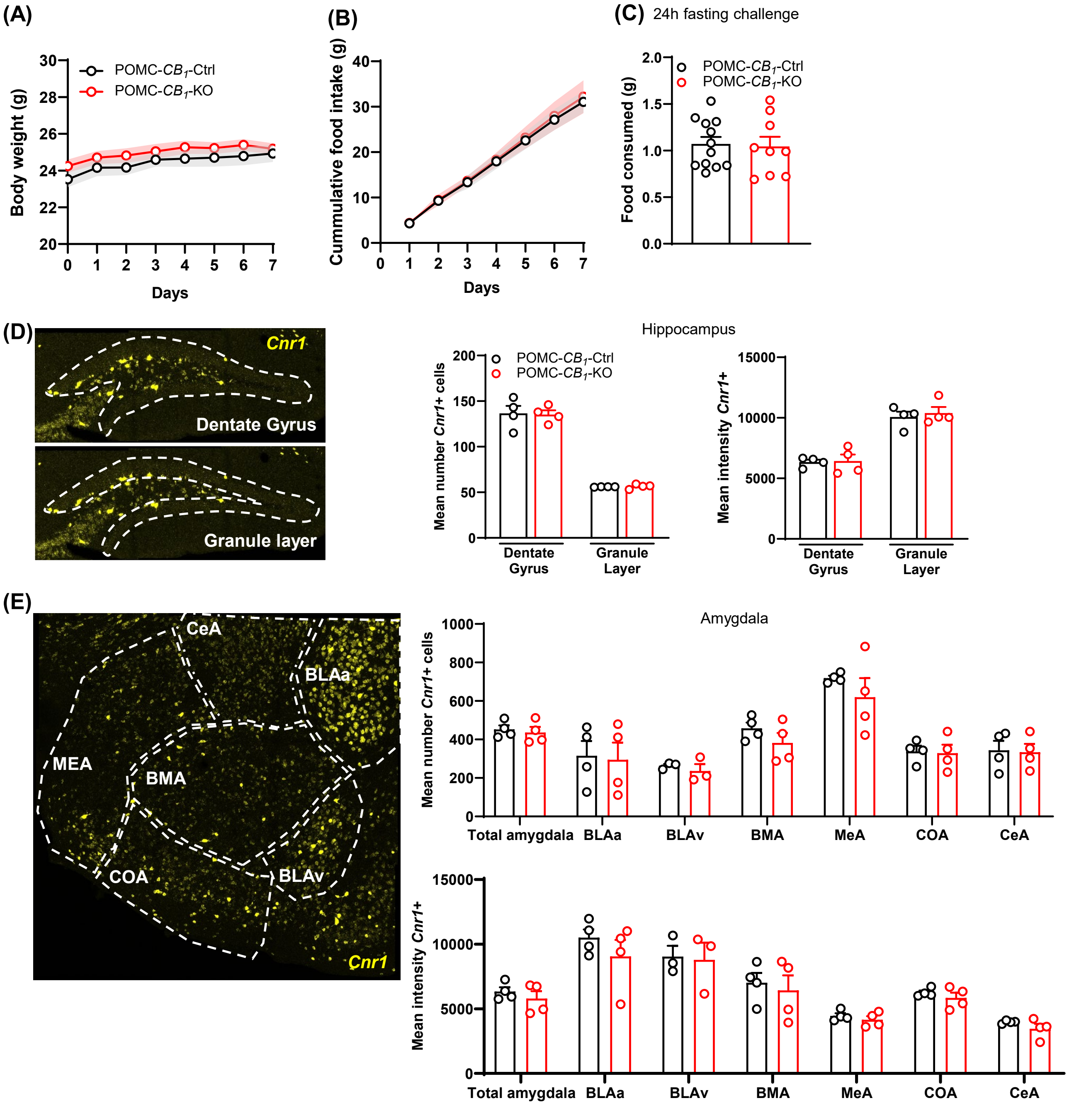


**Figure S4.** POMC-$CB_1$-KO mice and their control littermates showed similar (A) body weight, (B) food intake, and (C) 2h refeeding response after 24 h fasting (*n* = 9 - 12 mice/group). *Cnr1* expression in the dentate gyrus and granule cells' layers of the hippocampus (*n* = 4 mice/group) (D), and in the different nuclei of the amygdala (*n* = 3 - 4 mice/group) (E) was similar between POMC-$CB_1$-KO and their control littermates. Data are represented as the mean ± s.e.m.

# Figure S5

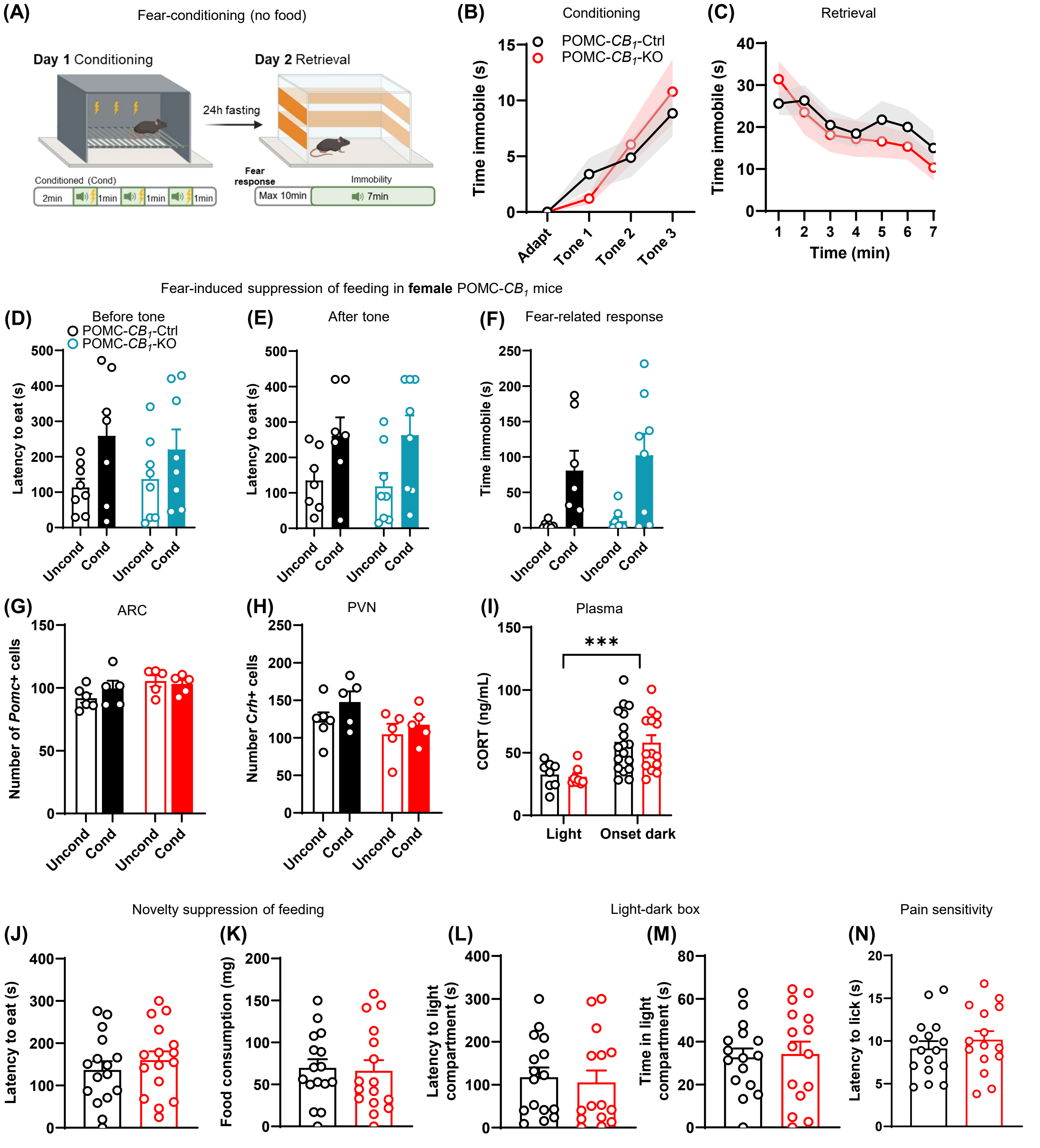

(A)
Fear-conditioning (no food)
Day 1 Conditioning
Day 2 Retrieval
24h fasting
Conditioned (Cond)
2min
1min
1min
1min
Fear response
Max 10min
Immobility
7min
(B)
Conditioning
POMC-CB1-Ctrl
POMC-CB1-KO
Time immobile (s)
Adapt
Tone 1
Tone 2
Tone 3
(C)
Retrieval
Time immobile (s)
Time (min)
Fear-induced suppression of feeding in female POMC-CB1 mice
(D)
Before tone
Latency to eat (s)
Uncond
Cond
(E)
After tone
(F)
Fear-related response
Time immobile (s)
(G)
ARC
Number of Pomc+ cells
(H)
PVN
Number Crh+ cells
(I)
Plasma
CORT (ng/mL)
***
Light
Onset dark
Novelty suppression of feeding
(J)
Latency to eat (s)
(K)
Food consumption (mg)
Light-dark box
(L)
Latency to light compartment (s)
(M)
Time in light compartment (s)
Pain sensitivity
(N)
Latency to lick (s)

**Figure S5.** Alterations in the fear-related response of conditioned POMC-$CB_1$-KO mice are specific to food presence since no differences were found between genotypes in fear-conditioning without food (A) on training (B) and retrieval (C) (*n* = 13 -16 mice/group). Female POMC-$CB_1$-KO displayed no genotype differences compared to their control littermates in the latency to eat before (D) or after the tone (E) and the fear-related response (F) in the fear-suppression of feeding (*n* = 7 - 8 mice/group). Both latency to eat after the tone (E) and fear responses (F) show statistically significant conditioning effect ($p<0.05$ and $p<0.0005$ respectively). (G) Number of *Pomc*+ cells in the ARC, and (H) *Crh*+ cells in the PVN were similar between groups and genotypes (*n* = 5 - 6 mice/group). (I) Corticosterone (CORT) plasma levels at the onset of the light phase and of the dark phase in non-stressful conditions were similar between control and POMC-$CB_1$-KO littermates (*n* = 8 - 18 mice/group, two-way ANOVA, genotype effect ***$P \leq 0.001$). In the novelty-suppressed feeding, a conflict-based test where hunger competes with an anxiogenic environment, POMC-$CB_1$-KO mice had similar latency to eat (J), and total food consumed (K) compared to their control littermates (*n* = 15 - 16 mice/group). (L) There were no differences between genotype in the latency to enter and time spent in the light compartment (M) of the light-dark box test (*n* = 15 - 16 mice/group). (N) POMC-$CB_1$-KO mice displayed pain sensitivity induced by heat similar to their control littermates (*n* = 15 - 16 mice/group). Data are represented as the mean ± s.e.m. Panel A was created using BioRender.com.
